# Cost-Effectiveness of Zoledronic Acid Versus Oral Alendronate for Postmenopausal Osteoporotic Women in China

**DOI:** 10.3389/fphar.2020.00456

**Published:** 2020-04-30

**Authors:** Ruxu You, Yu Zhang, David Bin-Chia Wu, Jinyu Liu, Xinyu Qian, Nan Luo, Takahiro Mori

**Affiliations:** ^1^Department of Pharmacy, Union Hospital, Tongji Medical College, Huazhong University of Science and Technology, Wuhan, China; ^2^School of Pharmacy, Monash University Malaysia, Bandar Sunway, Malaysia; ^3^Department of Pharmacy, Tongji Hospital, Tongji Medical College, Huazhong University of Science and Technology, Wuhan, China; ^4^Saw Swee Hock School of Public Health, National University of Singapore, Singapore, Singapore; ^5^Department of Health Services Research, Faculty of Medicine, University of Tsukuba, Tsukuba, Japan; ^6^Health Services Research and Development Center, University of Tsukuba, Tsukuba, Japan; ^7^Department of General Internal Medicine, Eastern Chiba Medical Center, Togane, Japan

**Keywords:** economic evaluations, Markov model, osteoporosis, fracture prevention, bisphosphonate

## Abstract

**Objective:**

This study aims to estimate the cost-effectiveness of yearly intravenous zoledronic acid treatment versus weekly oral alendronate for postmenopausal osteoporotic women in China.

**Methods:**

We used a Markov microsimulation model to compare the cost-effectiveness of zoledronic acid with alendronate in Chinese postmenopausal osteoporotic women with no fracture history at various ages of therapy initiation from health care payer perspective.

**Results:**

The incremental cost-effectiveness ratios (ICERs) for the zoledronic acid versus alendronate were $23,581/QALY at age 65 years, $17,367/QALY at age 70 years, $14,714/QALY at age 75 years, and $12,169/QALY at age 80 years, respectively. In deterministic sensitivity analyses, the study demonstrated that the two most impactful parameters were the annual cost of zoledronic acid and the relative risk of hip fracture with zoledronic acid. In probabilistic sensitivity analyses, the probabilities of zoledronic acid being cost-effective compared with alendronate were 70–100% at a willingness-to-pay of $29,340 per QALY.

**Conclusions:**

Among postmenopausal osteoporotic women in China, zoledronic acid therapy is cost-effective at all ages examined from health care payer perspective, compared with weekly oral alendronate. In addition, alendronate treatment is shown to be dominant for patients at ages 65 and 70 with full persistence. This study will help clinicians and policymakers make better decisions about the relative economic value of osteoporosis treatments in China.

## Introduction

Osteoporosis, characterized by bone mass reduction and microarchitectural deterioration of bone issue, has become a global public health concern worldwide (Cosman et al., 2014). Prevalence of osteoporosis in China has significantly increased over the past decade, from 14.94% before 2008 to 27.96% between 2012 and 2015 (Chen P. et al., 2016). This number is elevated for individuals of both genders above 50 years of age (34.65%), with postmenopausal osteoporosis being the most significant contributor (Tella and Gallagher, 2014; Chen P. et al., 2016). Postmenopausal osteoporotic can lead to hip, spine, wrist, and other fractures. These fractures significantly affect quality of life, work ability, and daily activities, and also increase financial burden through higher healthcare expenditure. Around 2.3 million osteoporotic fractures occurred in China in 2010 among people aged ≥ 50 years, costing US$9.5 billion; both the number and costs of osteoporosis-related fractures are estimated to double by 2035, reaching 6 million fractures costing US$25.4 billion by 2050 (Si et al., 2015; Liu et al., 2018).

A wide range of pharmacological treatments, such as commonly used bisphosphonates, have been shown to be effective in preventing osteoporotic fractures (Crandall et al., 2014; Sozen et al., 2017). However, effectiveness of oral alendronate is greatly reduced by poor persistence and compliance. Non-persistence, for example, happens commonly occurs between 42.5% and 80% of patients within 6 months (Hiligsmann et al., 2010; Recknor, 2011; Bianchi et al., 2015).

Zoledronic acid, once-yearly intravenous infusion, has become a popular alternative to oral alendronate for treating osteoporosis in postmenopausal women. Although systematic reviews with meta-analyses have shown zoledronic and alendronate to have similar efficiency on the reductions in the risks of different types of fractures (Murad et al., 2012; Zhou et al., 2016), there may exist a trade-off under investigation—zoledronic has a greater annual cost than alendronate but has also higher rates of persistence and compliance (Tremblay et al., 2016). While there are reports of zoledronic being a cost-effective treatment, they were conducted with Caucasian populations (Akehurst et al., 2011; Ito, 2018). Difference in epidemiology of osteoporotic fractures and healthcare system could render those results non-applicable in China.

To our knowledge, the economic value of zoledronic has not been assessed for Chinese women with postmenopausal osteoporosis. The current research aimed to analyse the pharmacoeconomic information of zoledronic acid compared to alendronate in Chinese postmenopausal women. The secondary aim of the study was to quantify the impact of medication persistence and compliance on economic evaluation.

## Methods

### Overview

The reporting of this current research followed the recommendations for the conduct of economic evaluations in osteoporosis (Hiligsmann et al., 2019). A Markov microsimulation model was previously built and validated to evaluate the cost-effectiveness of osteoporosis management in Japan and in the USA by one of our authors (Mori et al., 2017a; Mori et al., 2017b; Mori et al., 2019). The model was recently updated to compare the cost-effectiveness of zoledronic acid with alendronate in Chinese postmenopausal osteoporotic women with no fracture history at different ages of group initiation. The primary end point of this study was the incremental cost-effectiveness ratios (ICERs) expressed as cost per quality-adjusted life-years (QALYs) for one strategy compared with the other. The model aimed to simulate the entire lifetime of participants (up to 105 years old or until death) to capture relevant costs and consequences of fractures experienced during the treatment period. We estimated the cost-effectiveness from health care payer (only including direct medical costs) perspective. Costs and QALYs were discounted at an annual rate of 3% according to China Guidelines for Pharmacoeconomic Evaluations (Liu, 2011). Three times the per capita gross domestic product (GDP) value of China in 2018 ($29,340) was used as the willingness-to-pay (WTP) threshold. The economic modeling was developed in TreeAge Pro 2018 Software.

### Model Structure

**Figure 1:**
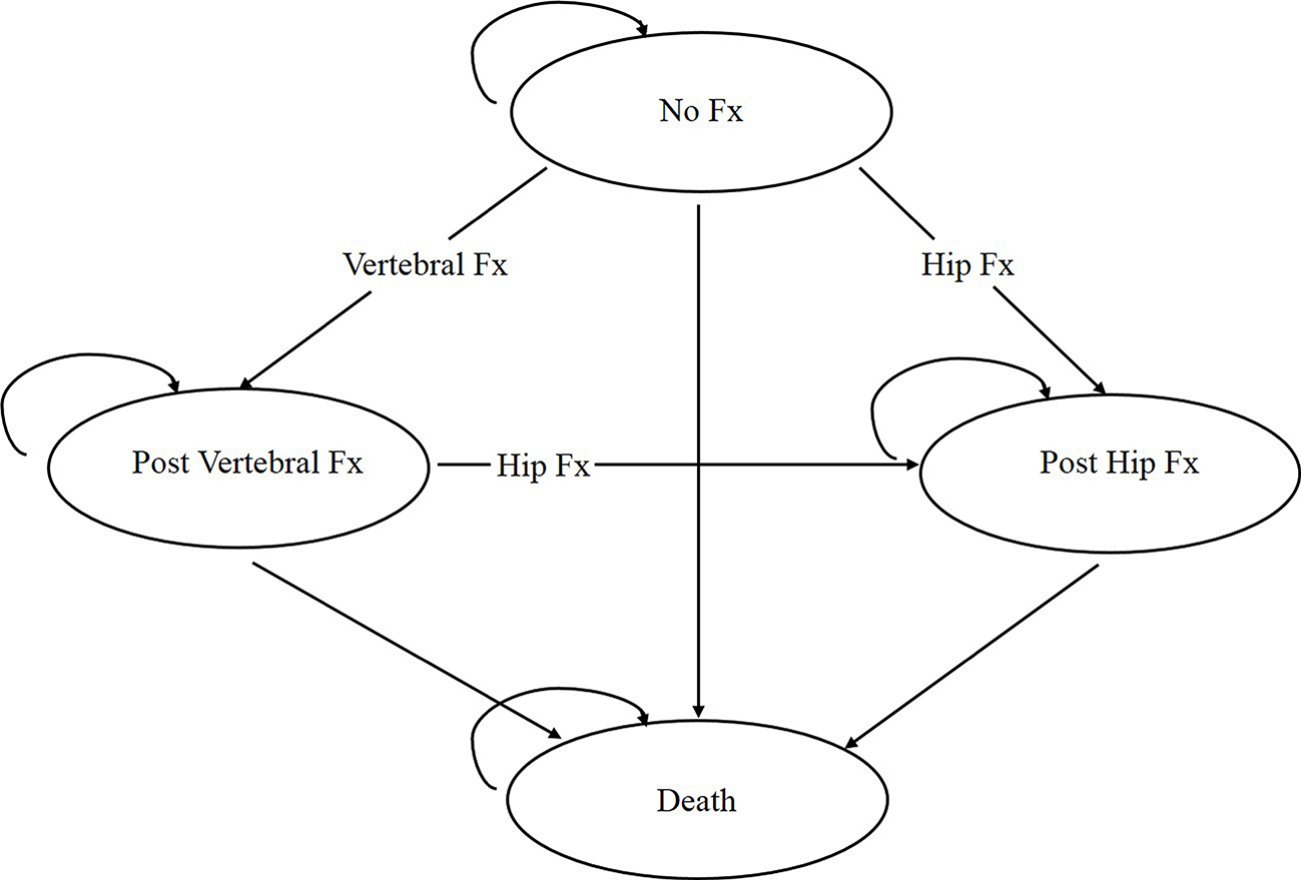
Structure of the osteoporosis state-transition model. Every participant starts the model in the “no fracture” state and transitions between health states or remains in the same states based on the assigned transition probabilities. Fx, Fracture.

The simplified representation of the model structure is shown in **Figure 1**. The individual begins the path in the “no fracture” state, and movement between states based on the transition probabilities already assigned. If a participant sustains a wrist or other osteoporotic fracture (i.e., humerus, distal forearm other than wrist, tibia/fibula, pelvis, or femur other than hip), corresponding one-time cost and disutility are assigned based on the Markov state the participant resides in. Tracker variables were created to record the number of each fracture type to adjust transition probabilities, costs, and utilities to reflect the impact of prior fractures. The length of a cycle in the model is 1 year as events rarely occur more than once a year (Mori et al., 2017a; Mori et al., 2017b; Mori et al., 2019). A participant can sustain only one fracture per cycle. A participant can have a maximum of two hip fractures but unlimited clinical vertebral, wrist, and other osteoporotic fractures over the entire time horizon. **Table 1** shows the key parameters used in the health economics model.

**Table 1:** Summary of key parameters in the model.

Parameter	Value	Range	Distribution	Reference	
Alendronate therapy					
Relative risk of hip fracture	0.45	0.27–0.68	Beta	(Murad et al., 2012)	
Relative risk of clinical vertebral fracture	0.50	0.33–0.79	Beta	(Murad et al., 2012)	
Relative risk of wrist fracture	0.50	0.34–0.73	Beta	(Wells et al., 2008)	
Relative risk of other fracture	0.78	0.66–0.92	Beta	(Murad et al., 2012)	
Persistence rate	0.57 (year 1)	N/A	N/A	(Johnell et al., 2005)	
Compliance	0.71 (year 1)	N/A	N/A	(Mori et al., 2017a)
Zoledronic acid therapy					
Relative risk of hip fracture	0.50	0.34–0.73	Beta	(Murad et al., 2012)	
Relative risk of clinical vertebral fracture	0.35	0.20–0.64	Beta	(Murad et al., 2012)	
Relative risk of wrist fracture	0.75	0.64–0.87	Beta	(Mori et al., 2017a)	
Relative risk of other fracture	0.69	0.55–0.84	Beta	(Murad et al., 2012)	
Persistence rate	0.73 (year 2)	N/A	N/A	(Tremblay et al., 2016)	
Costs (2018 US dollars)					
Annual cost for Alendronate	761.64	533.15–990.13	Triangular	(National Development And Reform Commission, 2018)	
Annual cost for Zoledronic acid	818.50	572.95–1,064.05	Triangular	(National Development And Reform Commission, 2018)	
Hip fracture, direct costs	7103.25	4972.28–9,234.23	Triangular	(Qu et al., 2014)	
Clinical vertebral fracture, direct costs	1,310.11	917.08–1,703.14	Triangular	(Qu et al., 2014)	
Wrist fracture, direct costs	967.34	677.14–1,257.54	Triangular	(Qu et al., 2014)	
Other fracture, direct costs	1,692.41	1,184.69–2,200.13	Triangular	(Qu et al., 2014)	
Annual cost for the post-hip fracture	4,438.08	3,106.66–5,769.50	Triangular	(Si et al., 2016)	
DXA scan	85	59.5–110.5	Triangular	(National Development And Reform Commission, 2018)	
Blood test	72	50.4–93.6	Triangular	(National Development And Reform Commission, 2018)	
Physician visit	10	7–13	Triangular	(National Development And Reform Commission, 2018)	
Utilities					
Age 65-69	0.806	0.765–0.846	Beta	(Sun et al., 2011)	
Age 70-74	0.747	0.709–0.784	Beta	(Sun et al., 2011)	
Age 75-79	0.731	0.694–0.767	Beta	(Sun et al., 2011)	
Age 80-84	0.699	0.664–0.733	Beta	(Sun et al., 2011)	
Age 85+	0.676	0.642–0.709	Beta	(Sun et al., 2011)	
Hip fracture, first year(multiplier)	0.776	0.720–0.844	Beta	(Si et al., 2014)	
Hip fracture, subsequent year(multiplier)	0.855	0.800–0.909	Beta	(Si et al., 2014)	
Clinical vertebral fracture, first year(multiplier)	0.724	0.667–0.779	Beta	(Si et al., 2014)	
Clinical vertebral fracture, subsequent year(multiplier)	0.868	0.827–0.922	Beta	(Si et al., 2014)	
Wrist fracture(multiplier)	0.940	0.910–0.960	Beta	(Hiligsmann et al., 2008)	
Other fracture(multiplier)	0.910	0.880–0.940	Beta	(Hiligsmann et al., 2008)	
Annual fracture incidence per 1,000 persons (without intervention)					
Hip fracture, age 65–69	0.96	N/A	N/A	(Wang et al., 2014)	
Hip fracture, age 70–74	2.33	N/A	N/A	(Wang et al., 2014)	
Hip fracture, age 75–79	4.08	N/A	N/A	(Wang et al., 2014)	
Hip fracture, age 80–84	6.44	N/A	N/A	(Wang et al., 2014)	
Hip fracture, age 85+	6.59	N/A	N/A	(Wang et al., 2014)	
Clinical vertebral fracture, age 65–69	5.64	N/A	N/A	(Bow et al., 2012)	
Clinical vertebral fracture, age 70–74	8.74	N/A	N/A	(Bow et al., 2012)	
Clinical vertebral fracture, age 75–79	12.05	N/A	N/A	(Bow et al., 2012)	
Clinical vertebral fracture, age 80–84	21.19	N/A	N/A	(Bow et al., 2012)	
Clinical vertebral fracture, age 85+	26.89	N/A	N/A	(Bow et al., 2012)	
Wrist fracture, age 65–69	12.95	N/A	N/A	(Lofthus et al., 2008)	
Wrist fracture, age 70–74	13.17	N/A	N/A	(Lofthus et al., 2008)	
Wrist fracture, age 75–79	13.87	N/A	N/A	(Lofthus et al., 2008)	
Wrist fracture, age 80–84	15.01	N/A	N/A	(Lofthus et al., 2008)	
Wrist fracture, age 85+	15.10	N/A	N/A	(Lofthus et al., 2008)	
Other osteoporotic fracture, age 65–69	6.60	N/A	N/A	(Mori et al., 2017b)	
Other osteoporotic fracture, age 70–74	9.84	N/A	N/A	(Mori et al., 2017b)	
Other osteoporotic fracture, age 75–79	14.44	N/A	N/A	(Mori et al., 2017b)	
Other osteoporotic fracture, age 80–84	18.06	N/A	N/A	(Mori et al., 2017b)	
Other osteoporotic fracture, age 85+	26.06	N/A	N/A	(Mori et al., 2017b)	
Relative risks of fractures for individuals with osteoporosis					
Hip fracture, age 65–69	3.91	3.28–4.56	Gamma	(Kanis et al., 2000; Johnell et al., 2005)	
Hip fracture, age 70–74	3.13	2.80–3.47	Gamma	(Kanis et al., 2000; Johnell et al., 2005)	
Hip fracture, age 75–79	2.60	2.39–2.82	Gamma	(Kanis et al., 2000; Johnell et al., 2005)	
Hip fracture, age 80–84	2.04	1.91–2.17	Gamma	(Kanis et al., 2000; Johnell et al., 2005)	
Hip fracture, age 85+	1.92	1.78–2.05	Gamma	(Kanis et al., 2000; Johnell et al., 2005)	
Clinical vertebral fracture, age 65–69	2.59	1.19–4.27	Gamma	(Marshall et al., 1996; Kanis et al., 2000)	
Clinical vertebral fracture, age 70–79	2.15	1.15–3.15	Gamma	(Marshall et al., 1996; Kanis et al., 2000)	
Clinical vertebral fracture, age 80+	1.82	1.12–2.41	Gamma	(Marshall et al., 1996; Kanis et al., 2000)	
Wrist fracture, age 65–69	1.78	1.78–2.19	Gamma	(Marshall et al., 1996; Kanis et al., 2000)	
Wrist fracture, age 70–79	1.6	1.60–1.88	Gamma	(Marshall et al., 1996; Kanis et al., 2000)	
Wrist fracture, age 80+	1.45	1.45–1.64	Gamma	(Marshall et al., 1996; Kanis et al., 2000)	
Other osteoporotic fracture, age 65–69	2.19	1.78–2.59	Gamma	(Marshall et al., 1996; Kanis et al., 2000)	
Other osteoporotic fracture, age 70–79	1.88	1.60–2.15	Gamma	(Marshall et al., 1996; Kanis et al., 2000)	
Other osteoporotic fracture, age 80+	1.64	1.45–1.82	Gamma	(Marshall et al., 1996; Kanis et al., 2000)	
Annual mortality rate					
65–69	0.01031	N/A	N/A	(Si et al., 2016)	
70–74	0.02036	N/A	N/A	(Si et al., 2016)	
75–79	0.03784	N/A	N/A	(Si et al., 2016)	
80–84	0.06998	N/A	N/A	(Si et al., 2016)	
85+	0.13603	N/A	N/A	(Si et al., 2016)	
Excess mortality after a hip fracture					
Relative hazard for mortality within a year after a hip fracture	2.87	2.52–3.27	N/A	(Haentjens et al., 2010)	
Relative hazard for mortality for second and beyond after a hip fracture	1.73	1.56–1.90	N/A	(Haentjens et al., 2010)	
Proportion of excess mortality after a hip fracture directly attributable to a hip fracture	0.25	N/A	N/A	(Kanis et al., 2003)	
Discounts					
Costs	0.03	0–0.05	Triangular	(Liu, 2011)	
Effectiveness	0.03	0–0.05	Triangular	(Liu, 2011)	

### Fracture Probabilities and Mortality

Annual hip and clinical vertebral fracture rates were based on recent epidemiological studies in the Chinese population (Bow et al., 2012; Wang et al., 2014). As incidence rates of wrist and other osteoporotic fractures are unavailable in the Chinese context, data were retrieved from papers in the USA and Norway (Melton et al., 1999; Lofthus et al., 2008). To accurately reflect the fracture risks of women with osteoporosis, the estimated values were adjusted using a method described in the previous works of one of our authors (Mori et al., 2017a; Mori et al., 2017b; Mori et al., 2019). The method calculated the relative risk of individuals below the threshold value (i.e. BMD T-score≤-2.5) compared with that of the general population (Kanis et al., 2000).

The age-specific background mortality rates were retrieved from the China Public Health Statistical Yearbook (National Health Committee of the People’s Republic of China, 2018). As described in the previous study, excess mortality was assumed following the hip fracture events (Haentjens et al., 2010). It is assumed that hip fracture events only contributed to 25% of the excess mortality, as comorbidities seem to play an important role (Kanis et al., 2003). No excess mortality was associated with clinical vertebral fractures (Mori et al., 2017a; Mori et al., 2017b; Mori et al., 2019).

### Treatment

Zoledronic acid strategy comprised 3 years of zoledronic acid at a dosage of 5 mg per year and alendronate strategy consisted of once-weekly oral alendronate therapy at a dosage of 70 mg per week. The relative risks of fractures on different treatments were obtained from the recent network meta-analysis (Gauthier et al., 2012). It was assumed that relative reductions of treatment were identical regardless of age and there was no difference in effectiveness between generic and brand-name medicines. It was also assumed that participants undergoing therapy went through a physician visit, dual-energy X-ray absorptiometry (DXA), and blood test per year, as recommended by the Chinese guidelines for the diagnosis and treatment of primary osteoporosis (Xia et al., 2019).

Persistence and compliance during the medical treatment were considered in our research (Stevenson and Selby, 2014; Hiligsmann et al., 2015). A higher compliance rates in the clinical therapy of oral alendronate than observational studies that reflected actual real world setting. The impact of their difference was included into the economic model by presuming a linear regression to assess relationship between the relative risk reduction and treatment compliance (Mori et al., 2017a; Mori et al., 2017b; Mori et al., 2019). Additionally, the residual benefits on fracture risk for those who discontinue treatment were considered, known as the offset-time effect. For those who discontinued therapy, no treatment effect was received and offset-time was assumed to be equal to the duration of their treatment period (Hiligsmann et al., 2012).

### Costs

Direct medical costs included the costs of drugs, fracture-related treatments, physician visits, DXA scans, blood testings, and long-term care costs associated with hip fracture. All costs were converted to 2018 USD using the web-based currency converter (CCEMG-EPPI-Centre Cost Converter, 2008).

The costs of alendronate and zoledronic acid were based on different brand prices and their respective market share in China. Medication costs were assumed to be proportional to their compliance and persistence with treatments (i.e., assuming compliance of alendronate therapy is 71% in the first year, the estimation of annual cost for alendronate is $761.64 × 0.71 in the first year). For individuals who discontinued alendronate within the first year, a 6-month fee was charged. We also included the medical expense for intravenous injection and the prevention of influenza-like symptoms into the expense of zoledronic acid.

Medical costs of the first year following fracture and annual long-term care costs for the “post-hip fracture” state were derived from previously published studies in Chinese setting (Qu et al., 2014; Si et al., 2016). Costs of physician visits, DXA scans, blood testings, and long-term care costs were sourced from the health system or the National Development and Reform Commission of China (National Development And Reform Commission, 2018).

### Utilities

Age-and sex-specific baseline health state utility values for those without osteoporotic fractures were obtained from the Chinese National Health Services Survey (Sun et al., 2011). We considered the relative reductions of utility attributable to the hip or clinical vertebral fractures, and the proportionate effects of a fracture on utility values in the first and subsequent year were derived from a recent meta-analysis (Si et al., 2014). Other osteoporotic fractures were assumed to only have a decreased risk of utility in the first year. (Hiligsmann et al., 2008).

#### Model Simulation and Sensitivity Analysis

For base case analyses, we ran the model with 100,000 iterations (100,000 individuals through the model one at a time) for individuals ages 65, 70, 75, and 80. Deterministic (one-way) sensitivity analyses were conducted by varying each key model parameter, while keeping all other variables constant at their base case values, over a range of values derived from 95% confidence intervals or the range informed in the relevant article. Probabilistic sensitivity analyses were performed to assess the effects of uncertainty in key model parameters simultaneously using Monte-Carlo simulations. In this method, all parameters were randomly drawn for 1,000 iterations from distributions of their probabilities and 10,000 trials per simulation. Three scenario analyses were carried out: (A) the patients with full persistence, (B) the patients with full compliance, and (C) the patients with both full persistence and full compliance.

## Results

### Model Validation

Consistent with data in the Chinese life table (National Health Committee of the People’s Republic of China, 2018), the economic model estimated that without intervention, the probabilities of dying by 105 years old were 99.0 (initial age 65 years), 98.8 (initial age 70 years), 98.5 (initial age 75 and 80 years), respectively. The model also predicted that without intervention, the lifetime probabilities of women experiencing at least one hip fracture or one clinical vertebral fracture by 65 years old were 11.099% and 39.693%, respectively, consistent with epidemiological data in China (Xia et al., 2019).

### Base Case Analysis

In the base case for postmenopausal osteoporotic women, the mean incremental costs and QALYs for zoledronic acid instead of alendronate were $1,014 and 0.043, $851 and 0.049, $824 and 0.056, and $718 and 0.059 at ages 65, 70, 75, and 80, respectively. The ICERs for the zoledronic acid versus alendronate were per QALY: $23,581 at age 65 years, $17,367 at age 70 years, $14,714 at age 75 years, and $12,169 at age 80 years, respectively. Compared with the alendronate strategy, the net monetary benefit (NMB) value of zoledronic acid ranged from 247.62 to 1,013.06, and the net health benefit (NHB) from 0.008 to 0.035 at different ages (**Table 2**).

**Table 2:** Base case results at various ages of therapy initiation.

	Cost (2018 USD)	ΔC	Effectiveness (QALYs)	ΔE	ICER (USD/QALY gained)	NMB	NHB
Aged 65 years							
Alendronate	10,572		12.755				
Zoledronic acid	11,586	1,014	12.798	0.043	23,581	247.62	0.008
Aged 70 years							
Alendronate	9,067		9.731				
Zoledronic acid	9,918	851	9.780	0.049	17,367	586.66	0.020
Aged 75 years							
Alendronate	7,245		7.329				
Zoledronic acid	8,069	824	7.385	0.056	14,714	819.04	0.028
Aged 80 years							
Alendronate	5,800		5.412				
Zoledronic acid	6,518	718	5.471	0.059	12,169	1,013.06	0.035

ICER, incremental cost-effectiveness ratio; NHB, net health benefit; NMB, net monetary benefit; QALYs, quality-adjusted life years; USD, United states Dollars; ΔC, incremental costs; ΔE, incremental effectiveness.

### Deterministic Sensitivity Analysis

**Figure 2:**
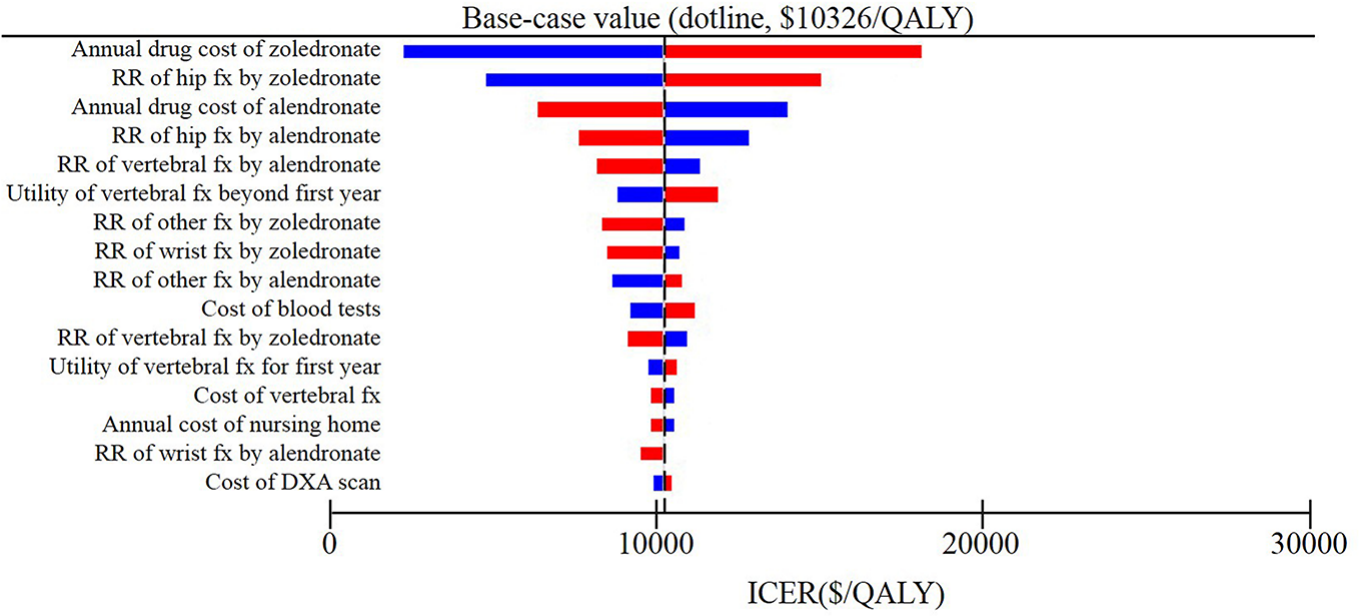
Results of deterministic sensitivity analyses, age 80 years. Tornado diagram shows the lower and upper values for the cost effectiveness ratio of the zoledronic acid strategy to the alendronate strategy.

The results of deterministic sensitivity analysis were illustrated as tornado plots showing the influences of extreme variations in each important parameter (**Figure 2** and **Supplemental Figures 1A–C**). Regardless of the starting ages, the study demonstrated that the two most impactful parameters were the annual cost of zoledronic acid and relative risk of hip fracture with zoledronic acid.

### Probabilistic Sensitivity Analysis

**Figure 3:**
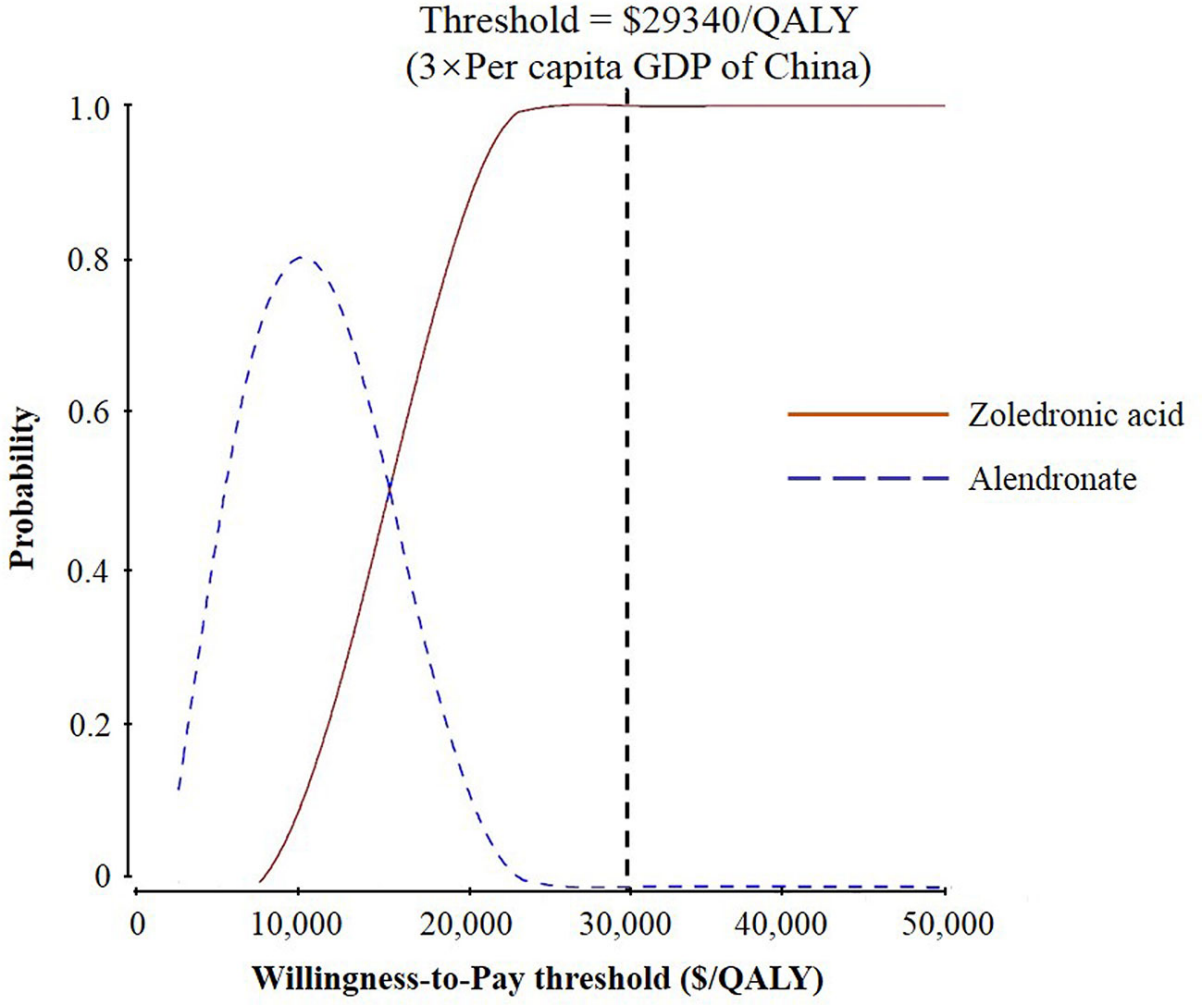
Results of probabilistic sensitivity analyses, age 80 years. The cost-effectiveness acceptability curves represent probabilities of being cost-effective achieved by the zoledronic acid strategy compared to the alendronate strategy at different willingness-to-pay (WTP) thresholds for postmenopausal osteoporotic women.

The results of probabilistic sensitivity analysis demonstrated that the probabilities of zoledronic acid being cost-effectiveness compared to alendronate were nearly 70, 81, 86, and 100% for starting ages 65, 70, 75, and 80 years, respectively, at a threshold of $29,340 per QALY (**Figure 3** and **Supplemental Figures 2A–C**).

### Scenario Analysis

**Figure 4:**
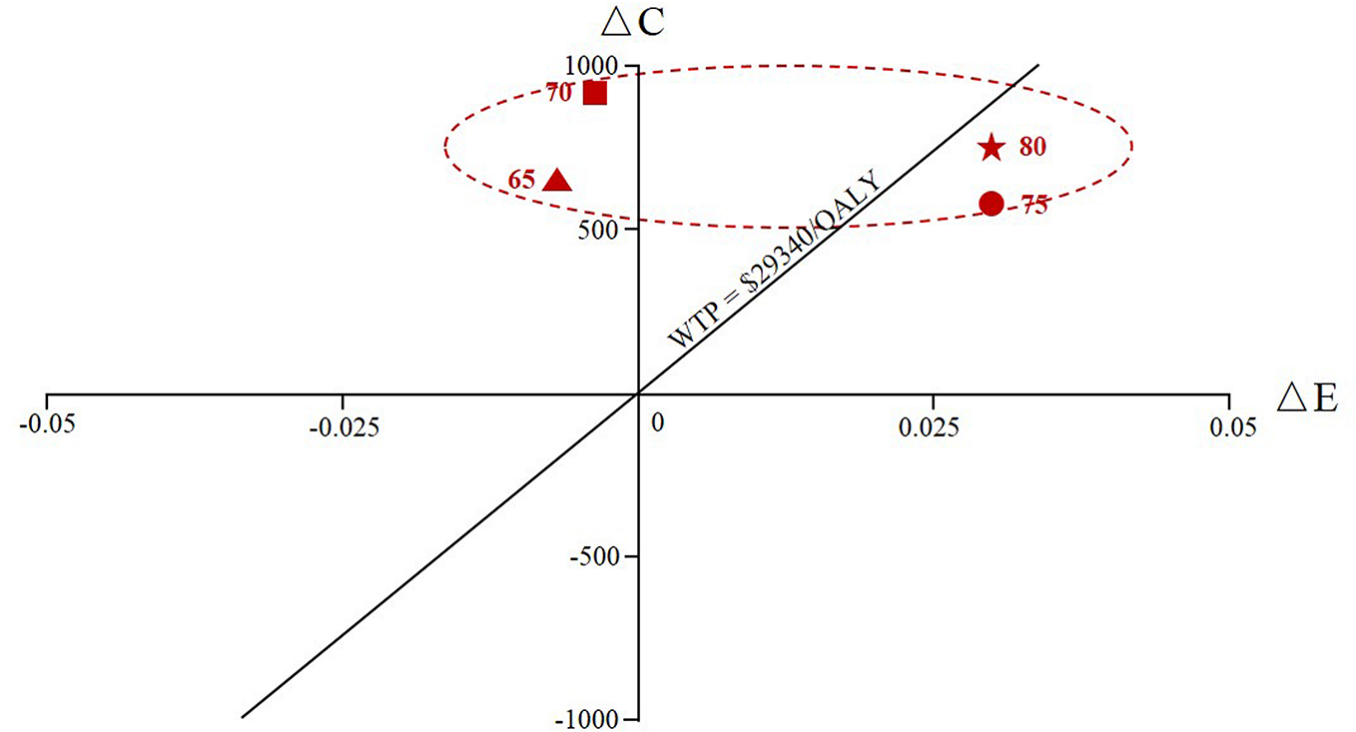
The cumulative cost and effectiveness of the zoledronic acid versus oral alendronate at various ages of therapy initiation (65, 70, 75, and 80) assuming alendronate therapy with full persistence. ΔC represented the incremental costs and ΔE represented the incremental effectiveness.

**Figure 4** and **Supplemental Figures 3A, B** indicated the outcome of the scenario analysis considering alendronate therapy persistence and compliance. If we assumed that the patients with full persistence, the results revealed that alendronate was found to be dominant (lower costs and greater QALYs) for patients at ages 65 and 70, while zoledronic acid was more cost-effective for ages 75 and 80 years given the current WTP threshold. If we assumed that the patients with full compliance, the ICER was ranged from 8,500 USD to 16,071 USD, making zoledronic acid cost-effective with a WTP of 29,340 USD per QALY gained. If simulated populations with both full persistence and full compliance, both costs and clinical effectiveness for alendronate treatment increased except at age 80 and the cost-effectiveness decision did not change.

## Discussion

In the current research, we evaluated the economic assessment for zoledronic acid treatment versus oral alendronate for postmenopausal osteoporotic women in China. Furthermore, we estimated the influences of alendronate persistence and compliance on clinical outcomes and costs. Base case results indicated that, compared with real-world data of alendronate, zoledronic acid therapy might be the optimal alternative option for simulated patients at all different starting ages examined at a threshold of $29,340 per QALY from health care payer perspective. In addition, alendronate treatment was shown to be dominant for patients at ages 65 and 70 with full persistence.

To the best of our knowledge, there are four studies reporting the cost-effectiveness of zoledronic acid for the treatment of osteoporosis. Ito K constructed a Markov model to compare the cost-effectiveness of single-dose zoledronic acid and supplementation of calcium and vitamin in the USA. In the base case, routine administration of zoledronic acid for nursing home residents with osteoporosis was not a cost-effective use of resource in the USA (Ito, 2018). In Japan, a patient-level state-transition model was designed to assess the cost-effectiveness of zoledronic acid versus alendronate for patients with osteoporosis who had a previous vertebral fracture. In the base case, they assumed 100% treatment persistence and compliance, which is not realistic in the real world. The results demonstrated that, although zoledronic acid was dominated by alendronate (i.e., less effective and more costly), considering the advantage of high compliance and persistence, zoledronic acid appeared a cost-effective treatment option (Moriwaki et al., 2017). Two other studies compared the cost-effectiveness of denosumab and zoledronic acid for elderly men with osteoporosis. The authors from the USA and Sweden concluded that denosumab was a cost-effective option for the treatment of elderly men (Parthan et al., 2014; Silverman et al., 2015).

Different methodological approaches, such as the model structure, time horizon and the measurements of costs and health utilities led to inconsistent findings of four published reports (Parthan et al., 2014; Silverman et al., 2015; Moriwaki et al., 2017; Ito, 2018). Compared to previous pharmacoeconomic analyses, the main differences of this research are the target population and comparator. The reason we choose postmenopausal osteoporosis is mainly based on the fact that most cases of osteoporosis occur in postmenopausal women, and the incidence increases with age in China (Lin et al., 2015; Chen P. et al., 2016). Administration of denosumab once every 6 months was not included as this therapy for osteoporosis was not marketed in China at the time of the current research.

Poor medication persistence and compliance are common problems of osteoporosis management, and they affect both effectiveness and cost of the treatments which are the key model parameters. Compared with weekly oral alendronate, our research found that the reason why zoledronic acid was cost effective was primarily due to zoledronic acid’s higher persistence rate. If we assumed that the patients had full persistence, the results revealed that alendronate was found to be dominant (i.e., lower costs and greater QALY) for patients at ages 65 and 70. It is worth noting that this heightened persistence rate of zoledronic acid was reinforced by our assumption of a residual effect from treatment; the risk for fracture returned to rates without treatment over the same years as the therapy duration in a gradual linear regression after stopping the treatment. This assumption has been consistently used in previous economic evaluations (Hiligsmann et al., 2012; Mori et al., 2017a; Mori et al., 2017b; Mori et al., 2019). Conversely, high compliance rate of zoledronic acid had a smaller effect than persistent rate on the results. This was mainly due to the fact that the compliance rate with weekly oral alendronate was already high.

The assumption of concurrent full persistence and compliance on the stimulated population, however, also directly increases medication costs. For example, average costs in the alendronate arm increased by 15.11% with full persistence and compliance compared with that in the base case at age 65 in the current study. While the assumption leads to overall improvements in clinical outcome, the improvement was found to be only marginal in this study. The average effectiveness with full persistence and compliance was 12.813 QALYs, which increased only by 0.45% relative to the base case. This is consistent with the previous study (Chen M. et al., 2016).

The results of the current economic model have to be interpreted within the context of some limitations. First, due to the heterogeneity of payer perspectives and the country-specific epidemiologic data used, our results should be conservatively generalized to women in other countries. Second, the current analysis did not take into account different osteoporosis screening strategies, which might have an impact on the cost effectiveness analysis. Third, although much of the data presented here to construct the model were obtained from Chinese data sets, some data were based on other countries, such as relative hazard for mortality after a hip fracture, proportion of excess mortality directly attributable to a hip fracture and incidence rates of wrist and other osteoporotic fractures. An updated pharmacoeconomic evaluation should be carried out when such data are available in the Chinese population. Finally, ever though we have identified for healthcare policymakers in China whether zoledronic acid treatment is of the best value for money, we have not attempted to consider issues of affordability (i.e., budget impact). This gap in research is an area for future research.

Our analysis may have several clinical and economic strengths. First, to the best of our knowledge, this is the first study to address the cost-effectiveness of zoledronic acid compared with oral alendronate based on a Chinese setting. The current study will provide valuable evidence to help health services researchers and policymakers guide policy formulation. Second, we integrated medication persistence and compliance into the health economic modeling and extensively tested how these changes in parameters have an influence on model outputs, as persistence and compliance have been considered to be important factors in economic evaluations for osteoporosis patients. Third, we used the Markov microsimulation modeling to track data separately by patient which avoids several restrictions of Markov cohort analysis that were widely used in pharmacoeconomic assessments. For example, patients with prior fractures might not be related with higher costs or probabilities subsequently in cohort model based on the “memoryless” feature. Since a microsimulation runs individual patients through the model, which fundamentally expands the researchers to be able to consider patient characteristics and prior events to affect the future values-probabilities, costs, and utilities, leading to a more realistic model and accurate results.

In conclusion, from the perspective of Chinese health care payer, once-yearly injection of zoledronic acid is estimated to be a cost-effective treatment option compared to weekly oral of alendronate for postmenopausal osteoporotic women without prior history of fracture at a threshold of 29,340 per QALY.

## Data Availability Statement

All datasets generated for this study are included in the article/**Supplementary Material**.

## Ethics Statement

Our study was approved in May 10, 2018 by the institutional ethics committee of Tongji Medical College of Huazhong University of Science and Technology, Wuhan, China. As this economic analysis was based on a literature review and modeling techniques, it was exempted from consent procedure by the institutional ethics committee of Tongji Medical College of Tongji Medical College of Huazhong University of Science and Technology.

## Author Contributions

Study design: RY and TM. Study conduct: RY and YZ. Data collection: JL. Data analysis: RY, YZ, and XQ. Data interpretation: RY, YZ, DW, NL, and TM. Drafting manuscript: RY and YZ. Revising manuscript content: RY, YZ, DW, JL, XQ, NL, and TM. Approving final version of manuscript: RY, YZ, DW, JL, XQ, NL, and TM. RY takes responsibility for the integrity of the data analysis.

## Funding

This work was supported by JSPS KAKENHI Grant Number JP17K08899.

## Conflict of Interest

TM: The joint appointment as an associate professor at the University of Tsukuba was sponsored by SMS Co., Ltd. in the fiscal year 2019 (i.e. April 2019 to March 2020). SMS Co., Ltd had no role in this study.

The remaining authors declare that the research was conducted in the absence of any commercial or financial relationships that could be construed as a potential conflict of interest.

## References

[B1] Akehurst R. Brereton N. Ariely R. Lusa T. Groot M. Boonen S. (2011). The cost effectiveness of zoledronic acid 5 mg for the management of postmenopausal osteoporosis in women with prior fractures: Evidence from Finland, Norway and the Netherlands. J. Med. Econ. 14, 53–64. 10.3111/13696998.2010.545563 21222506

[B2] Bianchi M. L. Duca P. Vai S. Guglielmi G. Viti R. Croci M. (2015). Improving adherence to and persistence with oral therapy of osteoporosis. Osteoporos Int. 26, 1629–1638. 10.1007/s00198-015-3038-9 25619634

[B3] Bow C. H. Cheung E. Cheung C. L. Xiao S. M. Loong C. Kung A. W. (2012). Ethnic difference of clinical vertebral fracture risk. Osteoporos Int. 23, 879–885. 10.1007/s00198-011-1627-9 21461720 PMC3277693

[B4] CCEMG-EPPI-Centre Cost Converter (2008). [Online]. Available: http://eppi.ioe.ac.uk/costconversion/default.aspx [Accessed July 18 2018].

[B5] Chen M. Si L. Winzenberg T. M. Gu J. Jiang Q. (2016). Cost-effectiveness of raloxifene in the treatment of osteoporosis in Chinese postmenopausal women: Impact of medication persistence and adherence. Patient Prefer. Adherence 10, 415–423. 10.2147/PPA.S100175 27099477 PMC4820231

[B6] Chen P. Li Z. Hu Y. (2016). Prevalence of osteoporosis in China: A meta-analysis and systematic review. BMC Public Health 16, 1039. 10.1186/s12889-016-3712-7 27716144 PMC5048652

[B7] Cosman F. de Beur S. J. LeBoff M. S. Lewiecki E. M. Tanner B. Lindsay R. (2014). Clinician’s guide to prevention and treatment of osteoporosis. Osteoporosis Int. 25, 2359–2381. 10.1007/s00198-014-2794-2 PMC4176573 25182228

[B8] Crandall C. J. Newberry S. J. Diamant A. Lim Y. W. Gellad W. F. Shekelle P. G. (2014). Comparative effectiveness of pharmacologic treatments to prevent fractures: An updated systematic review. Ann. Intern. Med. 161, 711–723. 10.7326/M14-0317 25199883

[B9] Gauthier K. Bai A. Perras C. Cunningham J. Ahuja T. Kovacs C. (2012). Denosumab, raloxifene, and zoledronic acid for the treatment of postmenopausal osteoporosis: Clinical effectiveness and harms (Ottawa (ON): Canadian Agency for Drugs and Technologies in Health). 24278999

[B10] Haentjens P. Magaziner J. Colon-Emeric C. S. Vanderschueren D. Milisen K. Boonen S. (2010). Meta-analysis: Excess mortality after hip fracture among older women and men. Ann. Intern. Med. 152, 380–390. 10.7326/0003-4819-152-6-201003160-00008 20231569 PMC3010729

[B11] Hiligsmann M. Ethgen O. Richy F. Reginster J. Y. (2008). Utility values associated with osteoporotic fracture: A systematic review of the literature. Calcif. Tissue Int. 82, 288–292. 10.1007/s00223-008-9117-6 18404243

[B12] Hiligsmann M. Rabenda V. Bruyere O. Reginster J. Y. (2010). The clinical and economic burden of non-adherence with oral bisphosphonates in osteoporotic patients. Health Policy 96, 170–177. 10.1016/j.healthpol.2010.01.014 20153543

[B13] Hiligsmann M. McGowan B. Bennett K. Barry M. Reginster J. Y. (2012). The clinical and economic burden of poor adherence and persistence with osteoporosis medications in Ireland. Value Health 15, 604–612. 10.1016/j.jval.2012.02.001 22867768

[B14] Hiligsmann M. Evers S. M. Ben S. W. Kanis J. A. Ramaekers B. Reginster J-Y. (2015). A systematic review of cost-effectiveness analyses of drugs for postmenopausal osteoporosis. Pharmacoeconomics 33, 205–224. 10.1007/s40273-014-0231-1 25377850

[B15] Hiligsmann M. Reginster J. Y. Tosteson A. Bukata S. V. Saag K. G. Lewiecki E. M. (2019). Recommendations for the conduct of economic evaluations in osteoporosis: Outcomes of an experts’ consensus meeting organized by the European Society for Clinical and Economic Aspects of Osteoporosis, Osteoarthritis and Musculoskeletal Diseases (ESCEO) and the US branch of the International Osteoporosis Foundation. Osteoporos Int. 30, 45–57. 10.1007/s00198-018-4744-x PMC6331734 30382319

[B16] Ito K. (2018). Cost-effectiveness of single-dose zoledronic acid for nursing home residents with osteoporosis in the USA. BMJ Open 8, e22585. 10.1136/bmjopen-2018-022585 PMC6129093 30181186

[B17] Johnell O. Kanis J. A. Johansson H. De Laet C. Delmas P. (2005). Predictive value of BMD for hip and other fractures. J. Bone Miner. Res. 20, 1185–1194. 10.1359/JBMR.050304 15940371

[B18] Kanis J. A. Johnell O. Oden A. Jonsson B. De Laet C. Dawson A. (2000). Risk of hip fracture according to the World Health Organization criteria for osteopenia and osteoporosis. Bone 27, 585–590. 10.1016/s8756-3282(00)00381-1 11062343

[B19] Kanis J. A. Oden A. Johnell O. De Laet C. Jonsson B. Oglesby A. K. (2003). The components of excess mortality after hip fracture. Bone 32, 468–473. 10.1016/s8756-3282(03)00061-9 12753862

[B20] Lin X. Xiong D. Peng Y. Q. Sheng Z. F. Wu X. Y. Liao E. Y. (2015). Epidemiology and management of osteoporosis in the People’s Republic of China: Current perspectives. Clin. Interv. Aging 10, 1017–1033. 10.2147/CIA.S54613 26150706 PMC4485798

[B21] Liu R. Chao A. Wang K. Wu J. (2018). Incidence and risk factors of medical complications and direct medical costs after osteoporotic fracture among patients in China. Arch. Osteoporos 13, 12. 10.1007/s11657-018-0429-5 29488018 PMC5829109

[B22] Liu G. (2011). China guidelines for pharmacoeconomic evaluations (Sciencepress: Beijing).

[B23] Lofthus C. M. Frihagen F. Meyer H. E. Nordsletten L. Melhuus K. Falch J. A. (2008). Epidemiology of distal forearm fractures in Oslo, Norway. Osteoporos Int. 19, 781–786. 10.1007/s00198-007-0499-5 17985071

[B24] Marshall D. Johnell O. Wedel H. (1996). Meta-analysis of how well measures of bone mineral density predict occurrence of osteoporotic fractures. BMJ 312, 1254–1259. 10.1136/bmj.312.7041.1254 8634613 PMC2351094

[B25] Melton L. R. Crowson C. S. O’Fallon W. M. (1999). Fracture incidence in Olmsted County, Minnesota: Comparison of urban with rural rates and changes in urban rates over time. Osteoporos Int. 9, 29–37. 10.1007/s001980050113 10367027

[B26] Mori T. Crandall C. J. Ganz D. A. (2017a). Cost-effectiveness of combined oral bisphosphonate therapy and falls prevention exercise for fracture prevention in the USA. Osteoporos Int. 28, 585–595. 10.1007/s00198-016-3772-7 27726000

[B27] Mori T. Crandall C. J. Ganz D. A. (2017b). Cost-effectiveness of denosumab versus oral alendronate for elderly osteoporotic women in Japan. Osteoporos Int. 28, 1733–1744. 10.1007/s00198-017-3940-4 28210776

[B28] Mori T. Crandall C. J. Ganz D. A. (2019). Cost-Effectiveness of Sequential Teriparatide/Alendronate Versus Alendronate-Alone Strategies in High-Risk Osteoporotic Women in the US: Analyzing the Impact of Generic/Biosimilar Teriparatide. JBMR Plus 3(11), e10233. 10.1002/jbm4.10233 31768491 PMC6874180

[B29] Moriwaki K. Mouri M. Hagino H. (2017). Cost-effectiveness analysis of once-yearly injection of zoledronic acid for the treatment of osteoporosis in Japan. Osteoporos Int. 28, 1939–1950. 10.1007/s00198-017-3973-8 28265718 PMC5486933

[B30] Murad M. H. Drake M. T. Mullan R. J. Mauck K. F. Stuart L. M. Montori V. M. (2012). Clinical review. Comparative effectiveness of drug treatments to prevent fragility fractures: A systematic review and network meta-analysis. J. Clin. Endocrinol. Metab. 97, 1871–1880. 10.1210/jc.2011-3060 22466336

[B31] National Development and Reform Commission (2018). [Online]. Available: http://www.ndrc.gov.cn/ [Accessed July 26 2018].

[B32] National Health Committee of the People’s Republic of China (2018). China health statistical yearbook. (Beijing: Peking Union Medical College Publishing House).

[B33] Parthan A. Kruse M. Agodoa I. Silverman S. Orwoll E. (2014). Denosumab: A cost-effective alternative for older men with osteoporosis from a Swedish payer perspective. Bone 59, 105–113. 10.1016/j.bone.2013.11.002 24231131

[B34] Qu B. Ma Y. Yan M. Wu H. H. Fan L. Hong Z. (2014). The economic burden of fracture patients with osteoporosis in western China. Osteoporos Int. 25, 1853–1860. 10.1007/s00198-014-2699-0 24691649

[B35] Recknor C. (2011). Zoledronic acid for prevention and treatment of osteoporosis. Expert Opin. Pharmacother. 12, 807–815. 10.1517/14656566.2011.562201 21385149

[B36] Si L. Winzenberg T. M. de Graaff B. Palmer A. J. (2014). A systematic review and meta-analysis of utility-based quality of life for osteoporosis-related conditions. Osteoporos Int. 25, 1987–1997. 10.1007/s00198-014-2636-2 24562840

[B37] Si L. Winzenberg T. M. Jiang Q. Chen M. Palmer A. J. (2015). Projection of osteoporosis-related fractures and costs in China: 2010-2050. Osteoporos Int. 26, 1929–1937. 10.1007/s00198-015-3093-2 25761729

[B38] Si L. Winzenberg T. M. Chen M. Jiang Q. Neil A. Palmer A. J. (2016). Screening for osteoporosis in Chinese post-menopausal women: A health economic modelling study. Osteoporos Int. 27, 2259–2269. 10.1007/s00198-016-3502-1 26815042

[B39] Silverman S. Agodoa I. Kruse M. Parthan A. Orwoll E. (2015). Denosumab for elderly men with osteoporosis: A Cost-Effectiveness analysis from the US payer perspective. J. Osteoporos 2015, 627631. 10.1155/2015/627631 26783494 PMC4689973

[B40] Sozen T. Ozisik L. Basaran N. C. (2017). An overview and management of osteoporosis. Eur. J. Rheumatol. 4, 46–56. 10.5152/eurjrheum.2016.048 28293453 PMC5335887

[B41] Stevenson M. D. Selby P. L. (2014). Modelling the cost effectiveness of interventions for osteoporosis: Issues to consider. Pharmacoeconomics 32, 735–743. 10.1007/s40273-014-0156-8 24715605

[B42] Sun S. Chen J. Johannesson M. Kind P. Xu L. Burstrom K. (2011). Population health status in China: EQ-5D results, by age, sex and socio-economic status, from the National Health Services Survey 2008. Qual. Life Res. 20, 309–320. 10.1007/s11136-010-9762-x 21042861 PMC3052443

[B43] Tella S. H. Gallagher J. C. (2014). Prevention and treatment of postmenopausal osteoporosis. J. Steroid Biochem. Mol. Biol. 142, 155–170. 10.1016/j.jsbmb.2013.09.008 24176761 PMC4187361

[B44] Tremblay E. Perreault S. Dorais M. (2016). Persistence with denosumab and zoledronic acid among older women: A population-based cohort study. Arch. Osteoporos 11, 30. 10.1007/s11657-016-0282-3 27679503

[B45] Wang J. Wang Y. Liu W. D. Wang F. Yin Z. S. (2014). Hip fractures in Hefei, China: The Hefei osteoporosis project. J. Bone Miner. Metab. 32, 206–214. 10.1007/s00774-013-0484-3 23784553

[B46] Wells G. Cranney A. Peterson J. Boucher M. Shea B. Coyle D. (2008). Risedronate for the primary and secondary prevention of osteoporotic fractures in postmenopausal women. Cochrane Database Syst. Rev. D4523. 10.1002/14651858.CD004523.pub3 18254053

[B47] Xia W. B. Zhang Z. L. Lin H. Jin X. L. Yu W. Fu Q. (2019). Guidelines for the diagnosis and management of primary osteoporosis. Chin. J. Osteoporos 25, 281–309. 10.3969/j.issn.1006-7108

[B48] Zhou J. Ma X. Wang T. Zhai S. (2016). Comparative efficacy of bisphosphonates in short-term fracture prevention for primary osteoporosis: A systematic review with network meta-analyses. Osteoporos Int. 27, 3289–3300. 10.1007/s00198-016-3654-z 27273112

