# Supplementary figures and images for: Cost-Effectiveness of Zoledronic Acid Versus Oral Alendronate for Postmenopausal Osteoporotic Women in China
Source: Front Pharmacol. 2020 Apr 30;11:456. doi: 10.3389/fphar.2020.00456 (PMC7203488; doi:10.3389/fphar.2020.00456)

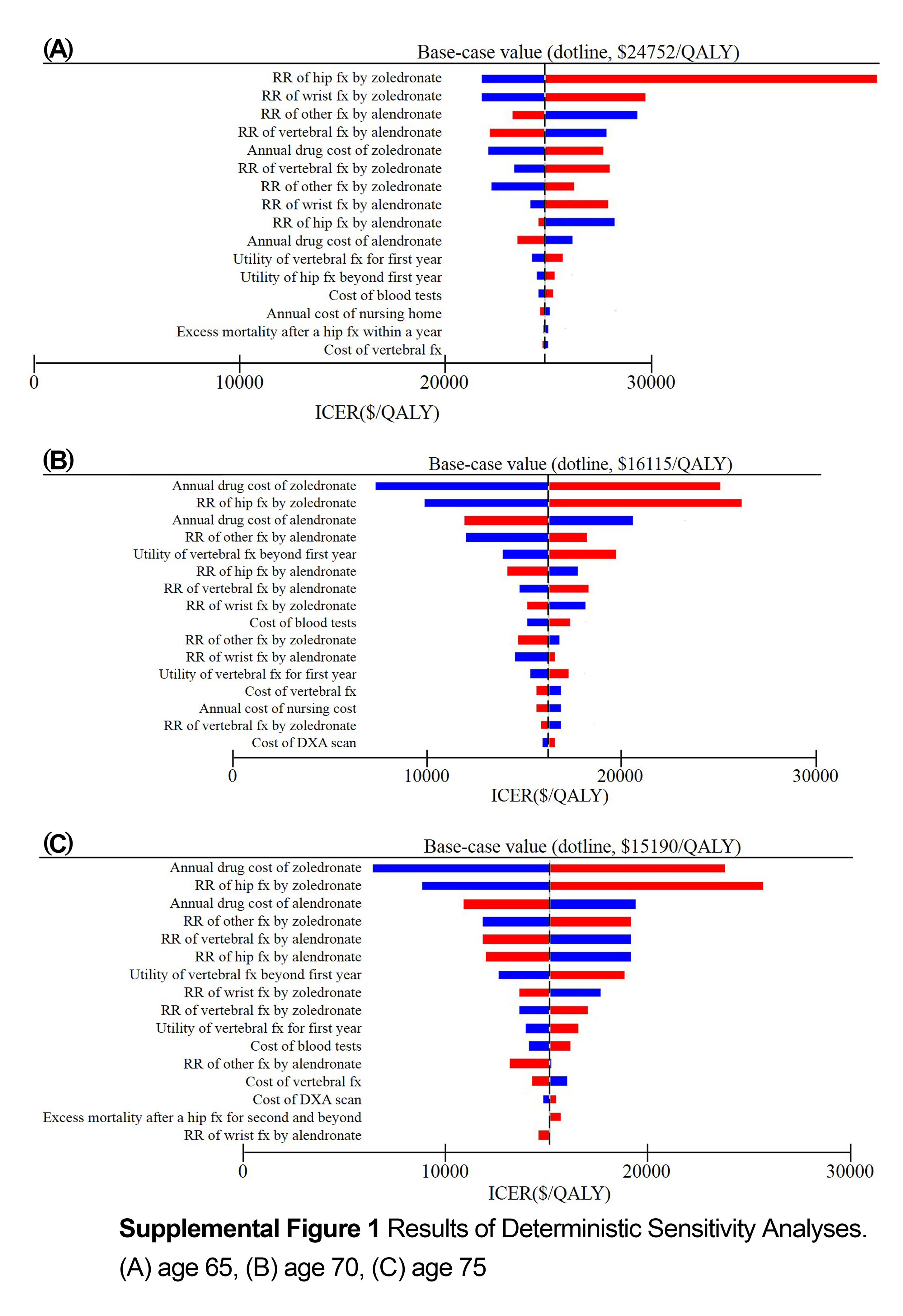

Supplement: Supplementary file 1 [file Image_1.tif]

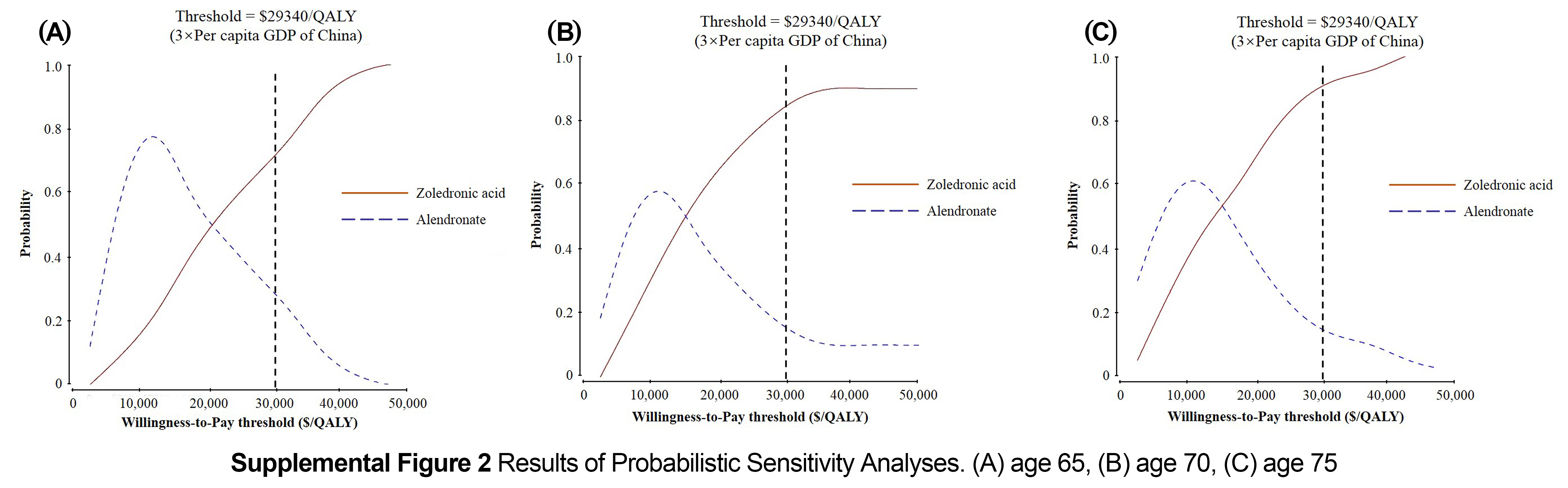

Supplement: Supplementary file 2 [file Image_2.tif]

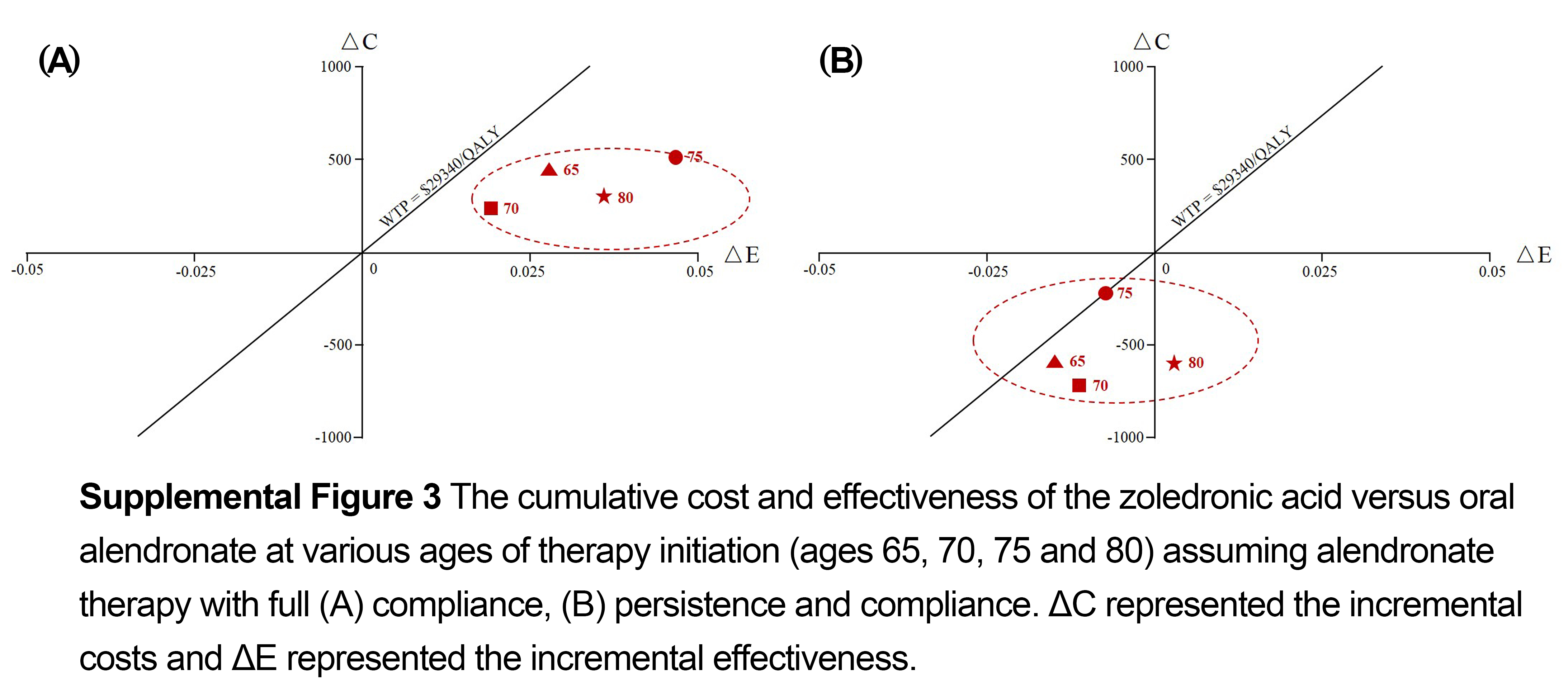

Supplement: Supplementary file 3 [file Image_3.tif]
